# Effect of food cues on time perception: influence of calories and diet control

**DOI:** 10.1038/s41598-022-24848-5

**Published:** 2022-11-25

**Authors:** Quentin Hallez, Lisa Filippone, Rebecca Shankland

**Affiliations:** 1 DIPHE, Institut de Psychologie, Université Lumière Lyon 2, 5 Avenue Pierre Mendès-France, 69500 Bron, France; 2 Institut Universitaire de France, Paris, France

**Keywords:** Human behaviour, Nutrition

## Abstract

The aim of this study was to investigate the influence on individuals’ time perception of observing a range of foods differing in calorific content. In a first experiment, 92 adult participants performed a temporal bisection task with stimulus durations presented in the form of high- or low-calorie food pictures as well as matched non-food control pictures. In a second experiment, 102 participants performed a strict replication of Experiment 1, without the low-calorie pictures condition as it showed less pronounced effects. Across the two experiments, the data revealed common results. An overestimation of time was observed in relation to high-calorie food pictures when compared with non-food pictures (Experiment 2), and the effect was a function of participants' diet control (Experiments 1 & 2). Contrary to our hypothesis, the more the participants reported controlling their diet, the less they overestimated the time when presented with food stimuli. The participants who controlled their diet reported being less aroused by the high-calorie food pictures, allowing the assumption that the modulation in time overestimation relies on the arousal response generated by high-calorie food pictures.

## Introduction

Temporal illusion can be defined as any difference between the time felt (i.e., subjective time) and the actual physical time, an objective and socially shared reference time measured by time measurement tools. The relative lengthening/shortening effects of perceived duration are an important part of our subjective reality because of the frequency of their daily occurrence. Among the various factors responsible for temporal illusion, researchers have particularly highlighted the role of emotions^1^.

Over the past few decades, research on time distortion in emotional contexts has greatly increased^2^. Researchers have demonstrated that exposure to emotional stimuli can lead individuals either to overestimate or underestimate time. These antagonist effects on perceived time depend on the characteristic of the prevailing emotional stimulus, namely arousal or attention^3,4^. Indeed, previous studies have shown that emotional stimuli which increase arousal levels generate a lengthening effect on perceived time duration^5–12^. Conversely, studies that have identified temporal underestimation effects have pointed to the influence of attentional factors. Numerous studies have shown a decrease in perceived time when attention is diverted away from the actual passing of time^13–20^.

These results can be explained in the light of the well-known pacemaker-accumulator model^21^, according to which every individual is equipped with an “internal clock”. The clock mechanism comprises a pacemaker, a switch, and an accumulator. At the onset of a stimulus to be timed, the switch closes, thereby allowing the pulses generated by the pacemaker to increment in the accumulator. At the offset of the stimulus, the switch opens and pulses can no longer transit to the accumulator. The perceived duration is thus directly dependent on the stored pulses, the perceived duration increasing as the pulses incrementally accumulate. This model remains the most commonly used due to its ability to explain the effects of emotional stimuli and the heuristic usefulness of the internal-clock metaphor^22^. It is important to highlight that other variants of this model exist, as well as other internal clocks^23^. Yet, none of these models has been unanimously approved^24^. According to the pacemaker accumulator model, increases in arousal modulate the functioning of the pacemaker by increasing its pulse emission frequency. As a result, more pulses will be produced and stored, thereby increasing the perceived time. Conversely, according to attentional models of time^20,25,26^, the more the attentional resources are diverted away from the passage of time, the more the perceived duration is shortened. A decrease in the attention allocated to the processing of time has the effect of increasing the latency relative to the closing of the switch, or of increasing its flickering during the duration processing (i.e., going from an open to a closed state).

Despite the emotional dimension that food can represent, little prior research has examined the emotional responses elicited by food stimuli. Only a small number of studies have employed pictures of food as emotional stimuli in the field of time perception^27–29^. In the majority of these studies, participants undertook a temporal bisection task. In this broadly-used task, participants are first asked to discriminate a short (S) from a long (L) standard duration, based on the presentation of a control picture (e.g., a white oval shape / empty plate). Then, in the testing phase, five intermediate durations are integrated along with the previous standard durations. In addition, emotional pictures (e.g., attractive or unattractive food) can be presented as temporal stimuli in addition to the previous control picture. The task remains unchanged for the participants, who must continue to judge the durations. The first results reported by Gil and colleagues^28^ showed an underestimation of time duration in relation to food pictures compared to control pictures, and that this underestimation was more pronounced for disliked food. The authors attributed this effect to an attentional bias mechanism, whereby food cues distract attention away from the processing of time. In contradiction to this attentional hypothesis, a recent meta-analysis^30^ conducted in non-clinical samples on a set of 57 datasets found little experimental evidence that greater dietary restriction is related to impaired cognitive control in general, or to increased cognitive bias for food. Thus, when examining specific attentional bias tasks, it appears that in healthy individuals without eating disorders, increased food restriction is not associated with increased attentional bias or distraction by food cues, regardless of the scale used to measure food restriction. Gagnon and colleagues^27^ showed no effect of food pictures on perceived duration in healthy participants, but they did demonstrate an overestimation effect from non-food to food cues in women with anorexia nervosa for both attractive and disgusting food. These authors noted that the effect on clinical samples may be related to experiential avoidance tendencies. To reduce their fear, women with anorexia nervosa rigidly avoid high-calorie food and are extremely reluctant to consume food outside a very narrow range^31,32^, which explains their reaction of greater fear. When these participants have no choice but to deal with foods, the situation raises their arousal level through an automatic response of the amygdala, which in turn speeds up the rhythm of their internal clock system^33,34^, which in turn increases the overestimation of time within this population.

To further investigate the influence of food stimuli on time perception, the present study aimed to investigate the influence of the calorific characteristics of foods (i.e., low-calorie and high-calorie) on time perception. Specific attention was also given to the daily diet control reported by participants. We assumed that their level of diet control would increase their fear reaction to food stimuli, similar to the recent study by Gagnon and colleagues^35^ on women with anorexia. Indeed, the more intense the emotion of fear elicited by a threatening cue, the more elevated the arousal response to it can be expected to be^36,37^. Thus, we expected that in the high-calorie picture condition compared to the non-food control picture condition, participants who control their diet would overestimate the time more than participants who do not control their diet, due to the fear reaction when confronted with calorific foods (based on the results reported by Gagnon and colleagues^27^). Conversely, we expected that in the low-calorie picture condition compared to the non-food picture condition, participants would underestimate time, independently of diet control levels, as no potential fear arises in this situation (based on the results of Gil and colleagues^28^). Furthermore, additional measurements were included on hunger and craving for food in order to be able to control for these variables, as it has previously been shown that they can affect arousal and/or valence^38–42^.

## Experiment 1

### Participants

A total of 92 participants took part in this experiment. As will be described more fully below, seven of them were removed from the database. The final sample therefore consisted of 85 participants, 68 of whom were females (80%). The participants’ age ranged from 18 to 27 years (*M* = 19.42, *SD* = 2.37). They were enrolled via an invitation communicated in the psychology courses of a French University and on social networks. The participants gave their consent via an electronic form presented at the beginning of the online experiment. The experiment was carried out in accordance with The Code of Ethics of the World Medical Association (Declaration of Helsinki) for experiments involving humans, and was approved by the research ethical committee of the University of Nantes.

### Material

**Figure 1:**
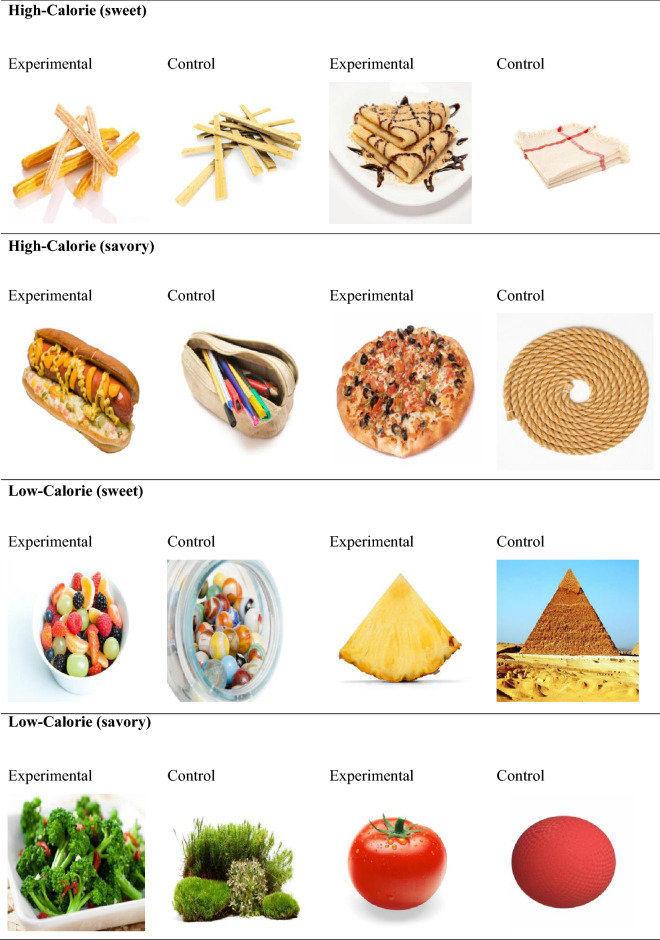
This Figure was designed by the authors of the present article and exposes the experimental pictures used in this study, paired with their control picture, for both high- and low-calorie foods associated with savory and sweet foods. The pictures, free of right, were taken from the FoodCal database^43^.

The survey was conducted using Qualtrics® electronic survey software, which recorded the participants’ responses and randomly delivered the stimuli. Three types of stimuli were presented: (1) four high-calorie food pictures (two sweet: crepes and churros, and two savoury: pizza and hotdogs); (2) four low-calorie food pictures (two sweet: pineapple and fruit salad, and two salted: tomato and broccoli); and (3) eight non-food pictures with similar characteristics in terms of shape, colours, and luminance to the non-food pictures (see Fig. 1). Two additional non-food pictures (of pink towels and a black box) were used for the training trials. All the pictures were taken from the Food-Cal database, which is a controlled database of high- and low-calorie food pictures matched with non‑food pictures^43^.

Two 100-point self-evaluation scales ranging from 0 (not at all) to 100 (absolutely) were used to measure (1) diet control; and (2) the participant’s hunger while performing the survey. The questions were as follows: (1) “I have the feeling that I control my diet in everyday life”, and (2) “At the time of completing this questionnaire, I am hungry”.

Finally, for each food picture, the participants were asked to complete three measures. The first two measures involved the completion of two dimensions (valence and arousal) of the Self-Assessment Manikin scale^44^. This affective rating system uses 9-point scales consisting of graphic illustrations to express how the participant feels while viewing the picture. Therefore, for the eight food pictures, the participants responded on two scales while imagining consuming the food item: one for the arousal dimension (from a sleepy to an excited figure), and the other for the valence dimension (from a frowning to a happy figure). The last measure was a 100-point self-evaluation scale ranging from 0 (not at all) to 100 (absolutely), which participants used to evaluate their desire to eat the food presented in the picture in the immediate future. The question associated with each food picture was as follows: “If I could, I would eat this food right away”. We called this measurement “craving” in our study.

### Procedure

The participants were first instructed not to eat in the two hours before the task, to take off their watch, to put their mobile phone away during the experiment, and to hide the clock on the menu bar of their computers to avoid any influence on time perception. They then performed a bisection task composed of three successive phases: pre-training, training, and testing. In the pre-training phase, the participants were exposed to the “short” (400 ms) and “long” (1600 ms) standard duration twice. The temporal stimuli took the form of control stimuli from the Food-Cal database. The participants were also asked not to count during the task, as this is an effective method used to prevent counting^45^, in order to better measure subjective time perception.

In the training phase, the participants were exposed to a block of 16 trials in which standard durations were each randomly presented eight times. Following each standard duration, the participants had to choose from responses labelled “short” or “long” displayed on the survey screen. They therefore had to discriminate between durations by ticking “short” after the short standard duration, or “long” after the long standard duration. They could not proceed to the next presentation without responding. Accuracy feedback was then presented in the centre of the screen for two seconds, with “yes” or “no” for correct and incorrect answers, respectively.

In the testing phase, the participants were asked to perform the same task, but the control stimuli were replaced by both control stimuli adapted to the food images used in our experiment, and by the food images themselves. Moreover, five intermediate durations (600; 800; 1000; 1200 and 1400 ms) were integrated in addition to the standard durations (400 and 1600 ms). Each food picture was displayed twice for each duration, making a total of 56 experimental trials for each of the high- and low-calorie picture conditions (4 food pictures × 2 repetitions × 7 durations = 56). There was also a third block of 56 trials, in which the eight non-food matched pictures were presented seven times each (once for each probe duration). In all, a total of 168 trials were randomly distributed.

### Statistical analyses

Data were analysed using SPSS v. 26 (IBM, 2019). First, descriptive statistics (i.t.o. means, standard deviations, skewness, kurtosis) and Pearson correlations were computed for each assessment interval to describe the data, the relationships between the factors, and to test for the assumptions underpinning the use of Repeated-measures analyses of variance (RM ANOVAs). Greenhouse–Geisser corrections were applied when violations of sphericity occurred.

The probability of a “long” response, P(long), was calculated for each individual in each picture condition for each duration in order to obtain psychophysical functions. In line with the traditional logarithmic four parameter sigmoid (L4P) model^46^, we fitted sigmoid functions to the calculated psychophysical curves. This method often provides excellent data fits and reasonable approximations of the BP and WR^47^. The data was modelled using Python. The BP is the “psychological mid-point” of the duration range between S and L, representing the duration producing 50% “short” responses and 50% “long” responses. Another parameter, the Weber Ratio (WR), was also calculated. The WR is an index of time sensitivity, and is the difference limen (p(long) = 0.75 – p(long) = 0.25) divided by the BP. A high WR indicates low time sensitivity, and a low WR indicates high time sensitivity. The program failed to fit an accurate sigmoid curve on seven psychophysical curves out of a total of 276 (*N*_High-Calorie_ = 1; *N*_Low-Calorie_ = 2; *N*_Control_ = 4). A total of seven participants were therefore rejected from our database. The data for both the WR and the BP did not violate the assumptions of multivariate normality (normal distribution), as absolute values of skewness and kurtosis did not exceed the + 2, to − 2 range threshold^48,49^. Additionally, all Mauchly’s sphericity tests being non-significant (*p* > 0.05), the variances of the difference between all combinations of the within- and between-group factors were equal (i.e., to test for an increase in Type 1 error).

Stimuli associated with trials for which the presentation duration was 200 ms longer than prescribed were deleted. We thus deleted 16 trials from a total of 14,280 trials, which corresponded to 0.11% of the data. On average, the presentation time compared to the expected time was accurate (*M* = 0.05 s; *SD* = 0.05 s). All the data are available on the following OSF link.

## Results

### Proportion of long responses

**Figure 2:**
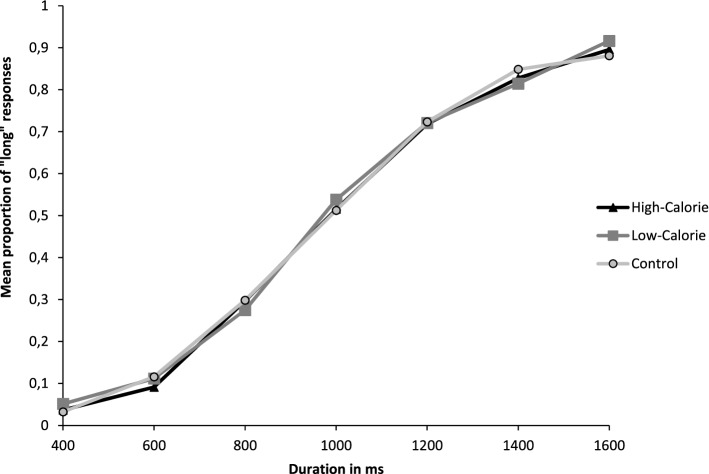
Mean proportion of “long” responses plotted against stimulus duration for each of the picture conditions.

Figure 2 shows the proportion of stimuli participants judged to be long (proportion of long responses, p(long)), plotted against the comparison durations for each picture condition.

A Repeated Measures Analysis of variance (RM-ANOVA) was launched on p(long) with the within-subject factors of picture condition (HC, LC and Control) and durations (400, 600, 800, 1000, 1200, 1400, 1600 ms). The RM ANOVA revealed a main effect of the duration *F*(6,79) = 885.03, *p* < 0.001; η^2^_p_ = 0.91. This suggests that the long response frequency is modulated by the duration of the stimulus. Additional paired t-tests corroborated this result, showing a significant increase in the long response rate between each of the seven durations (all *ps* < 0.001). The RM ANOVA did not reveal any main effect of the picture condition *F*(2,83) = 0.43, *p* = 0.65, nor a duration × picture condition interaction effect *F*(12,73) = 1.06, *p* = 0.39.

Yet, when the diet control variable was inserted as a covariate within the previous ANOVA, it substantially changed the results. First, the main effect of picture condition became significant *F*(2,81) = 3.17, *p* = 0.04. Also, the significant picture condition × control diet interaction *F*(2,81) = 4.29, *p* = 0.01; η^2^_p_ = 0.05 implies that the effect of the food pictures on time perception depends on the control that the individuals exerts over their diet. Apart from the main effect of duration which remains significant *F*(6,77) = 111.05, *p* < 0.001; η^2^_p_ = 0.57, the ANCOVA did not show any other significant effects (duration × diet control: *F*(6,77) = 0.96, *p* = 0.45; picture condition × duration: *F*(12,71) = 0.53, *p* = 0.90; picture condition × duration × diet control: *F*(12,71) = 0.46, *p* = 0.94).

**Figure 3:**
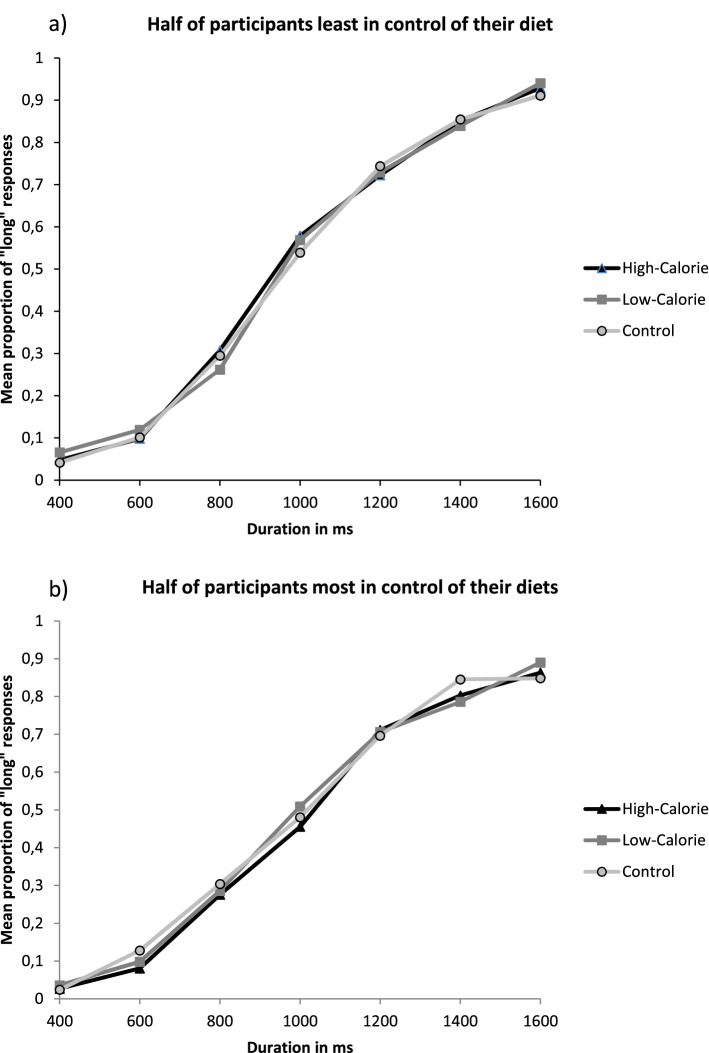
For graphical reasons only, this graph divides the participants according to their level of diet control. The upper panel shows the mean proportion of “long” responses plotted against stimulus duration for each picture condition associated with the half of the participants who control their diet least. The lower panel shows this same bisection task for the half of the participants who control their diet most.

**Figure 4:**
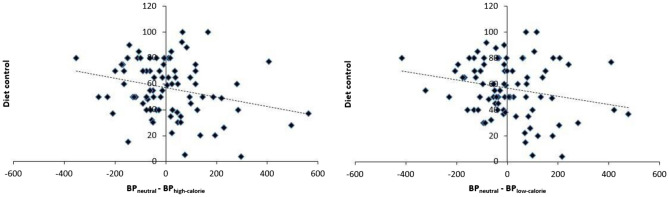
Graphical representation of the linear regression of the effect of diet control on the difference in time estimate between (left panel) BP_neutral_ and BP_high-calorie_ as well as (right panel) BP_neutral_ and BP_low-calorie_.

To examine differences in the perception of time as a function of the calorific characteristics of the food pictures, we conducted a series of RM ANOVAs on the proportion of long responses in order to perform a pairwise comparison between the different stimuli picture conditions. Figure 3 shows the proportion of stimuli participants judged to be long (proportion of long responses, p(long)), plotted against the comparison durations for each picture condition depending on participant’s control diet. In the case of high-calorie foods compared with the non-food stimuli, the RM ANOVA revealed a main effect of the picture condition *F*(1,82) = 5.36, *p* = 0.02; η^2^_p_ = 0.06, with a higher proportion of long responses associated with the control picture condition (*M* = 0.48, *SD* = 0.11) compared to the high-calorie picture condition (*M* = 0.49, *SD* = 0.11). The RM ANOVA also revealed a main effect of duration *F*(6,77) = 94.95, *p* < 0.001; η^2^_p_ = 0.54, as well as a significant picture condition × diet control *F*(1,82) = 7.28, *p* = 0.008; η^2^_p_ = 0.08. An additional regression analysis, illustrated in the Fig. 4, revealed that the lower the control the participants reported over their diet, the further they overestimated time (*t* = − 2.45, β = − 0.26, *SE* = 0.77, *p* = 0.02). In the case of low-calorie foods compared with the non-food stimuli, as well as for the low-calorie food compared with high-calorie pictures, there was no significant effect of the picture condition (*F*(1,82) = 2.14, *p* = 0.15 and *F*(1,82) = 1.36, *p* = 0.25, respectively), nor picture condition × diet control effect (*F*(1,82) = 2.17, *p* = 0.14; *F*(1,82) = 2.55, *p* = 0.11). It was notable that in all the performed ANCOVAs, all other interactions incorporating the duration variable failed to reach significance, as did the main effect of diet control (*F* < 2, *p* > 0.05).

### Psychophysical modelling

The RM ANOVAs with the within-factor of picture condition (high-calorie, low-calorie, non-food control) launched on the WR and the BP did not show any significant effect of the picture condition (BP: *F*(2,83) = 0.22, *p* = 0.80; WR: *F*(2,83) = 0.34, *p* = 0.71). The insertion of diet control as a covariate did not affect the results regarding the WR, which suggests that time sensitivity did not change according to the type of pictures presented. This result corroborates those of previous studies on emotions^2,50^. Yet, the latter has a drastic influence on BP. Indeed, the ANCOVA with the covariable of diet control revealed a main effect of the picture condition *F*(2,81) = 3.78, *p* = 0.02; η^2^_p_ = 0.04, as well as a significant picture condition × diet control interaction *F*(2,81) = 3.71, *p* = 0.03; η^2^_p_ = 0.04.

We therefore launched an ANCOVA series on the BP as a function of the calorific characteristics of the food pictures, with diet control as the covariable. The ANCOVA comparing high-calorie pictures with control ones revealed a main effect of the picture condition *F*(1,82) = 6.21, *p* = 0.01; η^2^_p_ = 0.07. This suggests that the 50% long response threshold arrives earlier in the high-calorie picture condition pictures compared to the control pictures (cf Table 1). In other words, the participants overestimate the time when presented with high-calorie pictures since they report a long percentage rate of 50% faster. It should be noted that this overestimation of time when faced with high-calorie food compared with the control pictures does not survive without the co-variable. Interestingly, the ANOVA also revealed a picture condition × diet control interaction *F*(1,82) = 6.02, *p* = 0.02; η^2^_p_ = 0.07; 1-β = 0.68. An additional regression analysis of the diet control variable on the difference from control to high-calorie pictures showed that the overestimation of time from control to high-calorie food decreases with the participant’s diet control (*t* = − 2.45, β = − 0.26, *SE* = 0.77, *p* = 0.02). The ANCOVA on the comparison between low-calorie food and non-food control pictures showed similar but less powerful effects. The main effect of picture condition *F*(1,82) = 3.99, *p* = 0.05; η^2^_p_ = 0.04, and the picture condition × diet control: *F*(1,82) = 4.04, *p* = 0.05; η^2^_p_ = 0.04, barely exceed the significance threshold, again with the lower BP values (e.g., overestimation) associated with the low calorie food pictures when compared to control pictures (cf Table 1), and a decrease in this overestimation with diet control (*t* = − 1.71, β = − 0.18, *SE* = 0.83, *p* = 0.09). Finally, the ANCOVA we ran on the two food picture conditions did not exhibit any main effect of picture condition *F*(1,82) = 0.41, *p* = 0.52, nor a picture condition × diet control interaction *F*(1,82) = 0.34, *p* = 0.56. Note that none of the ANCOVAs in the above section revealed diet control to have a main effect (F < 2, *ps* > 0.05).

**Table 1:** Descriptive statistics for the computed BP and WR associated with each picture condition.

	Mean	S.D	[Min;Max]	Skewness	Kurtosis
**High-Calorie**					
BP	987.6	171.3	[628;1466]	0.37	0.43
WR	0.40	0.20	[0.13;1.14]	1.12	1.58
**Low-Calorie**					
BP	993.9	174.7	[591;1502]	0.56	0.53
WR	0.40	0.21	[0.10;1.04]	0.97	0.28
**Control**					
BP	998.1	181.5	[609;1489]	0.23	− 0.32
WR	0.40	0.21	[0.11;1.04]	1.05	0.28

The statistical analyses performed on the WR did not show any significant results either (all *ps* > 0.05),

### Correlations between the high-calorie and non-food pictures and psychological measures

Table 2 shows the raw scores for the different psychological measures, as well as the differences between each variable and the calorie picture condition. In order to analyse the influence of each of these variables on the time distortion observed from the control to the experimental trials, the BP of the control condition was subtracted from the BP of the food condition (e.g., BP_Control_ – BP_Experimental_). Analyses were thus carried out to find correlations between this temporal distortion index and the variables of arousal, valence, and craving associated with the High- and Low-calorie conditions, as well as with hunger and the previously presented diet control variable for each of the food pictures. Table 3 shows the correlations found between each of the variables.

**Table 2:** Descriptive statistics for the arousal, valence, and craving measures associated with each condition of calorie foods.

Variables	High-Calorie foods			Low-Calorie foods			Differences between High and Low calorie foods				
	Mean	SD	[Min, Max]	Mean	SD	[Min, Max]	ΔMean (SE)	95% CI	t-Value	*p* value	Cohen’s d
Arousal	5.55	1.65	[2.00;8.50]	4.74	1.56	[1.00;7.75]	0.81(0.20)	[0.41;1.21]	4.07	< 0.001	0.39
Valence	6.84	1.31	[2.33;9.00]	6.12	1.49	[1.00;9.00]	0.72(0.20)	[0.33;1.11]	3.65	< 0.001	0.44
Crave	58.61	29.44	[0;100]	56.28	24.01	[1.25;100]	2.29(3.35)	[− 4.36;8.95]	0.69	.49	0.07

**Table 3:** Correlation matrix of the distortion observed on both p(long) over the three last durations and Bisection Points, as well as on the variables of Arousal, Valence, Craving, Hunger, and Control Diet, each of which are measures associated with the high and low control conditions.

Variables	HC					LC				
	1	2	3	4	5	1	2	3	4	5
1. BP distortion										
2. Arousal	− 0.01					0.00				
3. Valence	0.16	0.50**				0.03	0.15			
4. Craving	− 0.05	0.53**	0.58**			0.01	0.08	− 0.02		
5. Hunger	0.16	0.25*	0.26*	0.47**		0.03	− 0.07	− 0.08	0.47**	
6. Control diet	− 0.26*	− 0.20†	− 0.19†	− 0.16†	− 0.04	− 0.22*	− 0.22*	0.06	− 0.16	− 0.04

Note: †p < 0.01; *p < 0.05; **p < 0.01.

Contrary to our expectations, the effects of overestimation observed on BPs were not related to the arousal reported by the participants (HC: *r* = − 0.01, *p* = 0.92; LC: *r* = 0.00, *p* = 0.99). Positive correlations can be observed between Arousal, Valence, Hunger, and Craving (r > 0.20, *p* < 0.05).

Finally, the fact that diet control tended to correlate negatively with arousal (*r* = − 0.20, *p* = 0.07), valence (*r* = − 0.19, *p* = 0.08) and craving (r = − 0.20, *p* = 0.07) for high-calorie pictures indicates that it is the participants who control their diets the most that report lower arousal, valence, and craving effects when faced with high-calorie foods compared to participants who do not monitor their diet. The significant negative correlation between arousal and diet control on low-calorie foods pictures (*r* = − 0.22, *p* = 0.04) indicates that, more generally, individuals who exert control over their diet reported being less aroused by food cues.

### Discussion

Surprisingly, the results presented here invalidate all of our hypotheses. The analyses performed on the proportion of long responses and BPs as well as those on the BPs showed an overestimation of high-calorie food cues compared with non-food control pictures. In addition, this overestimation effect was directly dependent on the level of control participants reported that they had over their diet. The more the participants declared that they paid attention to their diet, the less they were victims of the time lengthening effect on BPs. The effects for low-calorie pictures were insignificant.

Interestingly, we found that diet control correlates negatively with arousal, valence, and craving. It would therefore seem that, contrary to our initial hypothesis, participants who control their diets react to a lesser extent to food cues. This effect could therefore be a way of explaining the reduced lengthening effect observed in participants controlling their diet. Indeed, the overestimation of the perceived time, a function of arousal as we presented it in the introduction, is also viable with positive valence stimuli^51^. Since non-diet-controlling individuals are further aroused by images of food, and all the more so by images of high-calorie foods, they show a less marked acceleration of the internal clock and therefore a lower overestimation effect, compared to their diet-controlling peers. This could also explain why low-calorie food pictures showed lower effects on time perception, because the pictures were less arousing than high-calorie food pictures. It therefore seems that all the results can be explained by a modulation of the arousal caused by food, which would consequently affect time perception.

To increase the robustness of these unexpected results, we decided to replicate our study. Since the effects were further pronounced for the high-calorie condition, we extracted the low-calorie condition from this replication. Our aim was to replicate (1) the overestimation of time caused by high-calorie pictures compared with control ones; and (2) to find an interaction between this overestimation and individual diet control levels, with lower overestimation effects associated with participants who control their diet more.

## Experiment 2

### Participants

A total of 109 participants performed this second experiment. The program failed to fit an accurate sigmoid curve for five of the participants, decreasing the total sample to 104 individuals, who ranged in age from 18 to 61 years (*M* = 29.15, *SD* = 13.66). This sample was made up of 91 females (87.5%), 11 males (10.5%), and 2 (2%) people who did not answer the gender question. The recruitment, including the ethical aspect, was similar to Experiment 1.

### Material and procedure

The material and procedure was similar to that used in Experiment 1, with the exception that the low-calorie condition was removed. As a consequence, the low-calorie pictures and the control pictures matched with the low-calorie pictures were withdrawn. The participants thus performed a total of 112 trials (2 pictures conditions × 4 pictures × 2 repetitions × 7 durations).

### Statistical analyses

A total of three reproductions were deleted from the database because they were associated with display durations that were 200 ms longer than the stimulus duration which should have been presented. This corresponded to 0.03% of the data (3 out of 11,648). On average, the presentation time compared to the expected time was accurate (*M* = 0.05; *SD* = 0.004).

The planned analyses were similar to those performed in study 1, and the preliminary analyses allowed us to perform RM ANOVAs. Nonetheless, at this point it is important to mention that Uni-ANOVA comparing the Experiment 1 group with the Experiment 2 group revealed that the populations differed in terms of reported arousal *F*(1,188) = 5.64, *p* = 0.02; η^2^_p_ = 0.03; 1-β = 0.66 (Experiment1: *M* = 5.55, *SD* = 1.66; Experiment 2: *M* = 4.98, *SD* = 1.68), valence *F*(1,188) = 17.68, *p* < 0.001; η^2^_p_ = 0.09; 1-β = 0.98 (Experiment1: *M* = 6.81, *SD* = 1.31; Experiment 2: *M* = 5.84, *SD* = 1.85), and diet control *F*(1,188) = 7.83, *p* = 0.006; η^2^_p_ = 0.04; 1-β = 0.79 (Experiment1: *M* = 56.75, *SD* = 21.61; Experiment 2: *M* = 46.07, *SD* = 29.08). The participants in this second experiment were also significantly older *F*(1,188) = 42.05, *p* < 0.001; η^2^_p_ = 0.18; 1-β = 0.18. Yet, no significant effects of the experiment were found, nor any interaction with the picture conditions (p > 0.05).

### Proportion of long responses

**Figure 5:**
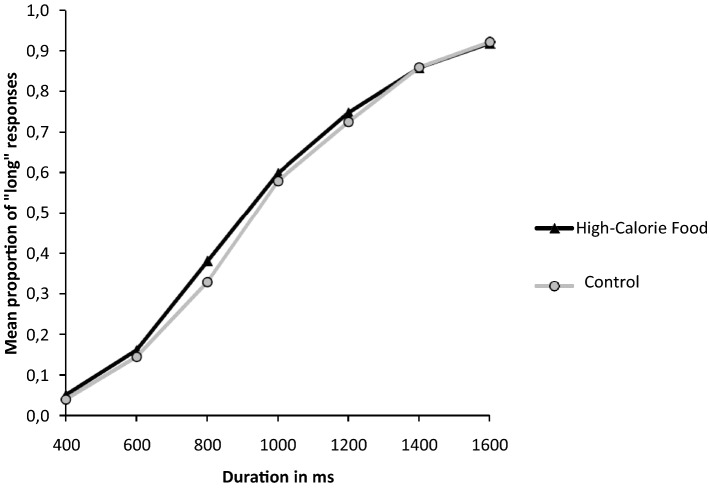
Mean proportion of “long” responses plotted against stimulus duration for high-calorie food and control picture conditions.

Figure 5 shows the proportion of stimuli participants judged to be long (proportion of long responses, p(long)) plotted against the comparison durations for each picture condition.

An RM-ANOVA was launched on p(long) with the within-subject factors of picture condition (HC, Control) and durations (400, 600, 800, 1000, 1200, 1400, 1600 ms). The model showed a main effect of duration *F*(6,98) = 855.43, *p* < 0.001; η^2^_p_ = 0.89, with a gradual increase in the long response rate between each of the seven durations (Bonferroni *ps* < 0.001). Unlike study 1, the ANOVA also disclosed a main effect of picture condition *F*(1,103) = 6.65, *p* = 0.01, η^2^_p_ = 0.06, with overestimation caused by high-calorie foods (*M* = 0.53, *SD* = 0.13) compared with the control pictures (*M* = 0.51, *SD* = 0.13). The lack of significance for the duration × picture condition interaction *F*(6,98) = 1.19, *p* = 0.31 implies that this overestimation is constant over time.

**Figure 6:**
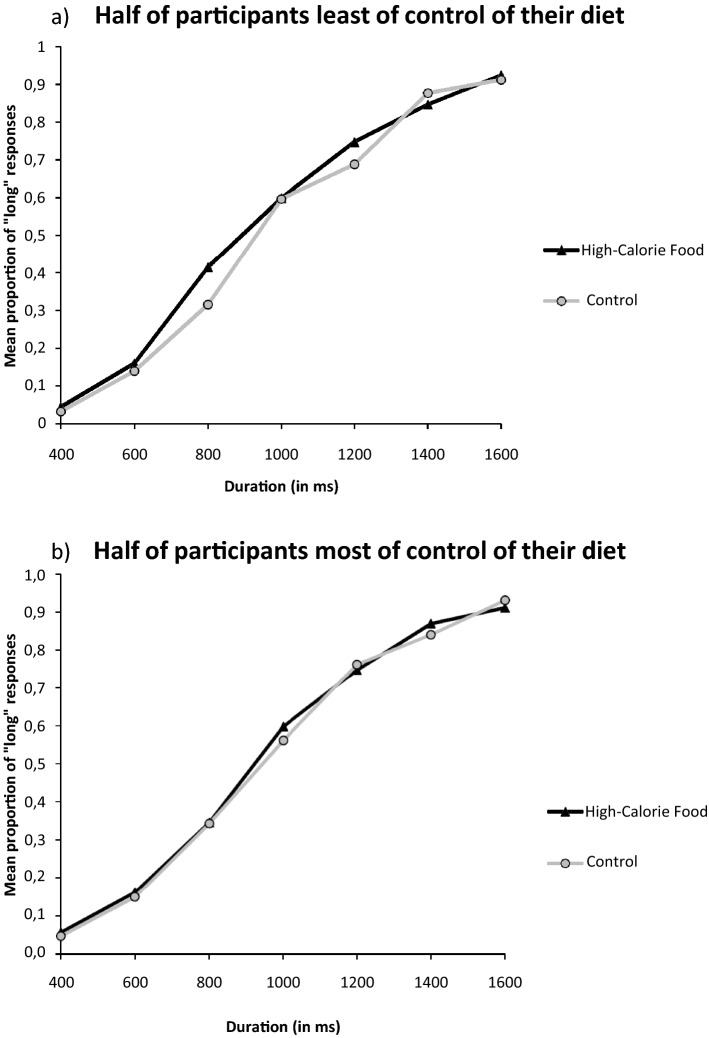
For graphical reasons only, this graph divides the participants of Experiment 2 according to their level of diet control. The upper panel shows the mean proportion of “long” responses plotted against stimulus duration for each picture condition associated with the half of the participants who control their diet least. The lower panel shows this same bisection task for the half of the participants who control their diet most.

**Figure 7:**
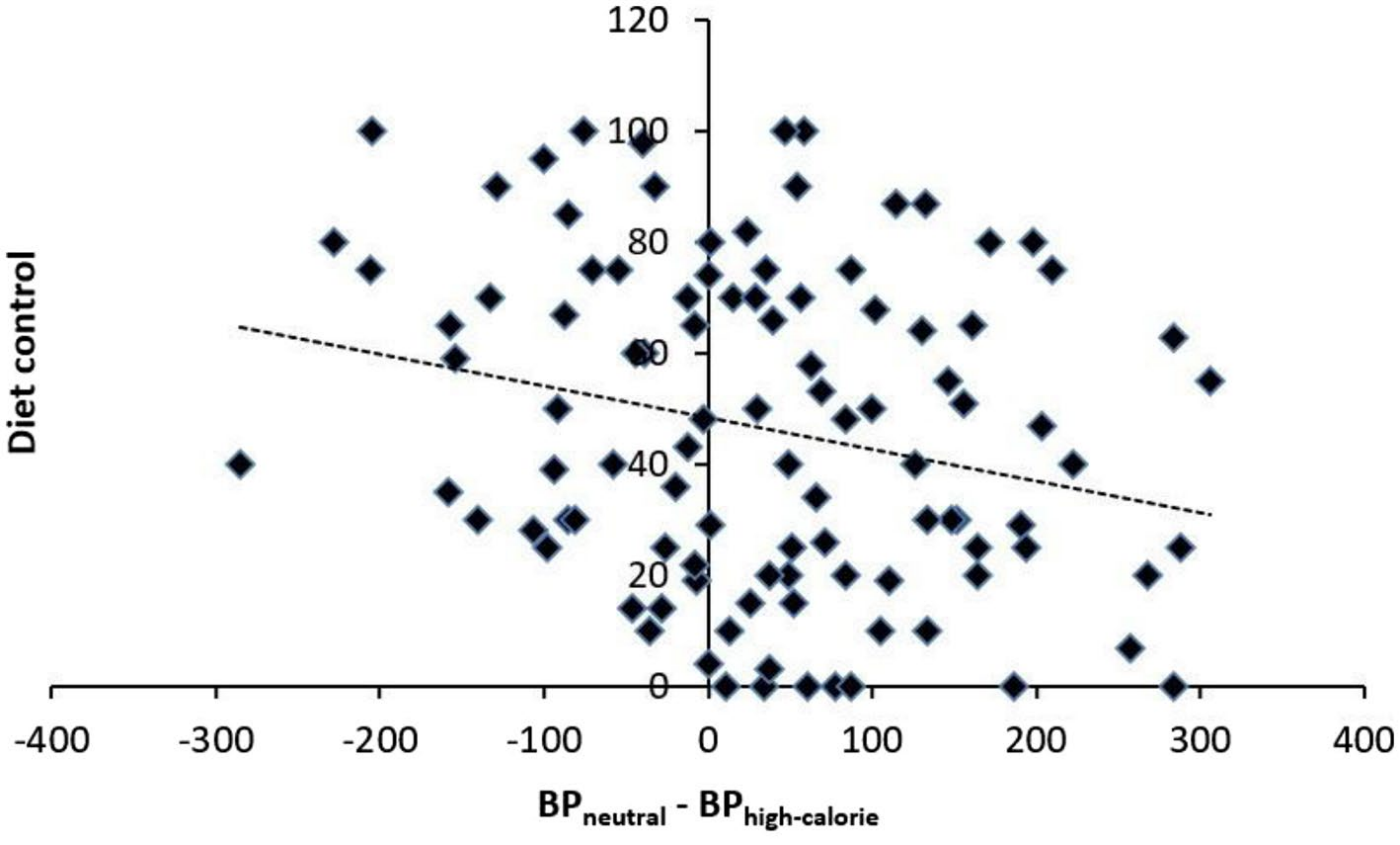
Graphical representation of the linear regression of the effect of diet control on the difference in time estimate between BP_neutral_ and BP_high-calorie_.

In order to analyse the possible influence of diet control over the main effect of the food pictures, we added the diet control variable as a covariable in previous models. Figure 6 illustrates the proportion of stimuli participants judged to be long (proportion of long responses, p(long)), plotted against the comparison durations for each picture condition depending on participant’s control diet. The main effect of the condition remained significant *F*(2,83) = 8.47, *p* = 0.004, η^2^_p_ = 0.03, but the picture condition × diet control, illustrated Fig. 7, was just a trend *F*(6,79) = 3.24, *p* = 0.07. Nonetheless, the model also showed a significant duration × picture condition × diet control (*F*(6,97) = 3.30, *p* = 0.003; η^2^_p_ = 0.03, suggesting that the modulation of one’s diet control on the effect of food pictures was different across the different durations.

When the ANCOVA was confined to the first four or five durations, the picture condition × duration × diet control interaction was not significant (*F*(3,100) = 2.37, *p* = 0.07; *F*(4,99) = 1.99, *p* = 0.09, respectively), suggesting that the interaction obtained with all seven durations was attributable to the convergence of the functions for food and non-food cues at the extreme long stimulus durations. Over the two ANCOVAs with longest durations, a significant interaction effect could be observed between the picture condition and the diet control (*F*(1,102) = 4.37, *p* = 0.04; η^2^_p_ = 0.04; *F*(1,102) = 8.84, *p* = 0.004; η^2^_p_ = 0.08, for the first four and five durations, respectively).

### Psychophysical modelling

Table 4 shows the computed BPs and WR associated with the high-calorie food and control pictures. An RM ANOVA was first launched with the within-factor of picture condition (high-calorie, non-food control) both on the WR and the BP. Jointly with the analysis launched on p(long), the ANOVA on the BPs showed a main effect of the picture condition *F*(1,102) = 9.66, *p* = 0.002, η^2^_p_ = 0.09, but did not reveal any effect on the WR *F*(1,84) = 3.07, *p* = 0.08. Inserting diet control as a covariate had no effect on the WR, but the results on the BP replicated the effects of Experiment 1. More specifically, the ANCOVA showed a significant picture condition × diet control interaction *F*(1,101) = 6.12, *p* = 0.01; η^2^_p_ = 0.06. An additional regression analysis of the diet control variable on the difference from control to high-calorie pictures was also performed. As has already been shown in Figs. 6 and 7, the results showed a decrease in the overestimation with diet control (*t* = − 2.47, β = − 0.24, *SE* = 0.40, *p* = 0.01). However, the main effect of the picture condition lost its significance *F*(1,101) = 0.46, *p* = 0.50, while the main effect of diet control remained non-significant *F*(1,101) = 0.48, *p* = 0.22.

**Table 4:** Descriptive statistics for the computed BP and WR associated with each picture condition for experiment 2.

	Mean	S.D	[Min;Max]	Skewness	Kurtosis
**High-Calorie**					
BP	929.4	200.61	[502;1413]	0.21	− 0.16
WR	0.42	0.17	[0.12;1.03]	0.70	0.59
**Control**					
BP	964.2	193.54	[512;1493]	− 0.01	0.03
WR	0.40	0.19	[0.11;1.12]	1.03	1.85

### Correlations between the high-calorie and non-food pictures and psychological measures

The correlation analysis series performed in Experiment 1 was replicated in this Experiment, and the results are shown in Table 5.

**Table 5:** Correlation matrix of the distortion observed on p(long) differences over the three last durations and Bisection Points, on Arousal, Valence, Craving, Hunger, and Diet Control.

Variable	1	2	3	4	5
1. BP distortion					
2. Arousal	0.21*				
3. Valence	− 0.01	0.36**			
4. Craving	0.01	0.38***	0.67**		
5. Hunger	0.09	0.43**	0.41**	0.51**	
6. Control diet	− 0.24*	0.02	− 0.10	0.11	− 0.02

Note: †p < 0.01; *p < 0.05; **p < 0.01.

A difference to be noted here is the significant positive correlation between arousal and the difference between control and high-calorie BP (*r* = 0.21, *p* = 0.03). This is an important result, as it could suggest that increased arousal might lead to a higher overestimation effect on the BPs, in line with internal clock models. Unfortunately, this second experiment did not show any correlation effects between the control diet and arousal (r = 0.02, *p* = 0.80), valence (r = − 0.10, *p* = 0.29), or craving (r = 0.11, *p* = 0.27).

### Discussion

The results for Experiment 2 replicate those of Experiment 1, making clearer the influence of exposure to high calorie foods on time perception. Indeed, in this experiment, the statistics on the proportion of long responses showed a significant effect of the picture condition, even though the diet control variable was not inserted as a covariate (in Experiment 1, the main effect was not observed without the co-variate of diet control).

This overestimation was, once again (both in the proportion of long responses and BPs) a function of the control that participants exert on their diets: specifically, the more that participants control their diet, the less they overestimate the time in the middle of the curve. Conversely, participants who do not report that they regulate their diet (or do so to a lesser extent) show a more marked overestimation effect.

### Ethics approval

The experiment was carried out in accordance with The Code of Ethics of the World Medical Association (Declaration of Helsinki) for experiments involving humans and was approved by the research ethical committee of the University of Nantes. Informed consent was obtained from all participants.

## General discussion

The aim of this paper was to analyse the emotional influence of food pictures on the time perception of healthy adults in relation to their diet control. In the first experiment, we experimentally manipulated the calorific characteristics of foods on time perception. The second experiment aimed at replicating the results of Experiment 1, focusing more on the effect of the high-calorie condition, since the low-calorie condition did not show significant effects.

Through the different studies, an effect of overestimation caused by exposure to high-calorie pictures and without any other variable was only observed in Experiment 2. This is therefore in line with the results of Gagnon and colleagues^27^, who found no effect of food pictures on the perception of time in healthy participants. The overestimation effect observed in this paper seems intrinsically linked to diet control, because the overestimation effect was systematically revealed when the diet control co-variable was integrated in the model. It was then revealed, throughout these two experiments, that BPs further shifted to the left for both low and high-calorie food pictures compared to control pictures. Probably the most intriguing result of this study is that this overestimation was a function of diet control, in that the more the participants reported controlling their diet, the less time was overestimated. Conversely, the less the individuals reported controlling their diet, the more they overestimated time.

What needs to be discussed now is the nature of such effects. Gil and colleagues^28^ found that the presentation duration of food pictures was underestimated compared with the presentation duration of control pictures, and that this underestimation was more marked for the disliked food pictures than for the liked ones. This underestimation was explained by the authors as being due to an attentional mechanism, in that food cues distract attention away from the processing of time more than control pictures do. As we set out in the introduction, viewed from a pacemaker-accumulator (internal-clock) interpretation of time processing^21,52^, with a reduction in the attentional resources allocated to time processing, either the pacemaker generates more oscillation between an open and closed state, or the switch closing latency is delayed. Given that time estimation relies on the number of impulses incremented within the accumulator, a loss of pulses produces a relative temporal underestimation^53,54^. It would therefore be legitimate to ask why we did not obtain the same underestimation effect. Firstly, it is important to note that Gil et al.^28^ did not consider the calorific content of the food presented (and also that the main focus of their study was to test disliked and disgusting food stimuli, which may explain the difference of effects). We can hypothesise that individuals do not treat all foods in the same way depending on the attraction or rejection that they may cause. For example, it has been shown that high-calorie foods are responsible for a dopamine release, even in the absence of homeostatic need^55^. However, a growing number of studies show a direct link between dopamine, an excitatory neurotransmitter, and our perception of time^56^. Dopaminergic neurons in the ventral periaqueductal gray (vPAG) are located at the crosstalk of two arousal routes of the ascending reticular activation system (ARAS), and can project to the hypothalamus via the dopamine (DA) receptor system^57^. Dopamine is therefore an excitatory neurotransmitter that increases arousal levels.

According to internal clock models, an increase in perceived time may be the consequence of an increase in the individual in question’s state of arousal. The more an individual is aroused, the more their internal clock races and emits a faster flow of impulses. Consequently, more time units are accumulated and the time is perceived as longer. This phenomenon has been demonstrated many times with negative or positive valence stimuli^2,51^. The observed temporal overestimation could therefore be linked to an increase in the state of arousal of the individuals being shown food pictures, which speeds up their internal clock. This is completely in line with the prior literature showing an increased state of arousal when individuals are faced with food cues^38,58^. The fact that in our study we found fewer temporal distortions in the condition of exposure to low-calorie foods can be understood in this way, as this type of stimuli is not very attractive and therefore not sufficiently excitatory. Indeed, the results show significantly lower arousal effects related with the low-calorie content compared to the high-calorie images.

The fact that this overestimation effect is reduced by the control that participants exert on their diet is also in favour of this arousal hypothesis. Indeed, in Experiment 1, participants who controlled their diets showed lower arousal and craving effects when faced with images of high-calorie foods. This result reflects dietary restraint, which can be defined as an actual reduction of food intake which relies on less pleasure and lower craving for both low- and high-calorie foods^59^. Dietary restraint is linked to reduced sensitivity to the hedonic and motivational value of food, regardless of calorific content, thereby matching the lower arousal of individuals reporting more diet control. It is therefore likely that a population which controls their diet well is more prone to limit certain food groups, such as fatty and/or sugary foods, for example. The consequence of the lower arousal state shown by individuals controlling their diet would thus be a diminished effect of temporal overestimation. The aforementioned links between diet control and reduction in arousal/craving were not replicated in Experiment 2, but a direct positive correlation was shown between the BP and arousal. This difference in diet control is reflected in the perception of time: the overestimation effect is higher in Experiment 2, where the individuals controlled their diet the least.

The fact that the effect of food was a function of diet control is modulated on the different durations (e.g., interaction duration × picture condition × diet control) also supports the arousal hypothesis. If the effect of time distortions was related to attention, then it should be constant over time^60^. Nonetheless, we cannot totally exclude the attentional hypothesis proposed by Gil and colleagues^28^, since the overestimation effect found in Experiment 1 was continuous over time. Moreover, no direct link was found between arousal and BP, and we observed that individuals who exerted the highest control over their diet even seemed to tend to underestimate time in the presence of high-calorie foods.

Although of interest, these results have some limitations. A first limitation relates to the measure of diet control. When considering the results, it may be unclear whether we were measuring restrictive eating—which should lead to greater arousal as suggested by the literature on eating disorders and in particular bulimia and binge eating^61–63^—or whether we were measuring self-regulation abilities^64,65^. Indeed, it appears that certain executive functions, such as updating, inhibiting, shifting, and working memory capacities, successfully contribute to the self-regulation of eating behaviors^66^. When considering the results of our study, we may suppose that it is not restrictive eating which has been measured by this item but self-regulation skills, and this may explain why diet control is not associated with greater arousal but with lower arousal. Participants who are high in self-regulation abilities may feel more comfortable (less aroused) when looking at high-calorie foods, as they are not afraid of losing control or feeling high levels of craving for that food. In this case, participants who report lower control of their diet would, in turn, have weaker self-regulation skills, which could therefore result in more arousal because they know that they can snap easily. Studies should therefore be carried out to analyse this possibility in more depth, and to categorise the results according to the different types of dietary restrictions. The fact that our study was based on a single diet control question instead of a validated questionnaire therefore does not allow us to answer this question. On the other hand, it should also be highlighted that studies have already validated the psychometric qualities of a single-item measure^67^ and other studies have shown that single-item measures are not necessarily associated with poor psychometric properties^68^.

In addition, using a questionnaire would have provided information on several of the sub-dimensions underlying diet control. Indeed, in a recent paper, Polivy et al.^69^ explained that although restricted eaters all share the goal of losing weight, they differ in their personalities, levels or types of motivation, ability, behaviours, attitudes, self-images and self-esteem, as well as in why they to want to diet. For these authors, this conceptual ambiguity must be recognised when talking about restricted and unrestricted eaters. For example, the Three Factor Eating Questionnaire^70^ and the Dutch Eating Behavior Questionnaire^71^ define dieters as individuals who are able to consistently restrict their food intake, and who are therefore more likely to lose or at least maintain their weight over time^72,73^. However, the Restraint Scale^74^ and the EDE-Q restrained eating subscale^75^ define dieters as individuals who try to restrict themselves but are often prone to disrupting their inhibitions then giving in to the temptation to eat more. These individuals generally fail to lose or even maintain their weight^76^. This distinction is thus between "successful" versus "unsuccessful" restriction. In a recent commentary on the Polivy et al.^69^ article, Lowe^77^ challenges the conceptualisation of restrained eating as a trait. Thus, for this author, dieting is not the cause of dietary abnormalities such as the deregulation of hunger and satiety responses, but rather, it is the consequence of an obesogenic environment that underlies a chronic susceptibility to overeating, weight gain, and loss of dietary control.

In future studies, it would be interesting to make additional measurements in order to measure the weight of other variables that have been shown to be important in the food psychology literature. Body Mass Index (BMI) is considered to be a health measure, and its use is only recommended in online research studies if it is essential to the study. As this was a first study on the topic linking the perception of time and food cues, we decided not to include this measure on this first occasion, but to do so in future studies based on specific hypotheses that will be formulated. Yet, it should be mentioned that researchers recently presented the positive relationship between the Body Mass Index and attentional biases^78,79^ where people who were overweight or obese manifested a greater top-down attention bias towards food cues, compared to people with a normal weight. Attentional resources having been shown to improve time distortion^13–20^, the possible effect of BMI on the biases of temporal perception of food should be explored. Along the same line, future studies should replicate these results within a lab-design study. Ambient atmospheres, including explicit or implicit visual, auditory, and olfactory stimulations, are known to have a significant impact on time sensitivity^80–84^. The small effect sizes reported in our studies could reflect this environmental noise.

In conclusion, our studies provide new findings about the influence of calories on emotions and time perception. For the first time, we have shown that food cues, and even more specifically high-calorie foods, were less overestimated in participants with higher levels of reported diet control. This has been linked to their reduced arousal reaction when presented with high-calorie foods. The observed reduction in arousal would therefore be very adaptive for individuals paying attention to their diet since they would have the feeling, at least implicitly, of being less confronted with highly calorific foods they do not wish to consume.

## Data Availability

The data that support the findings of this study are openly available at: https://osf.io/3vgm6/?view_only=17c557248d4a47d8a41ce100e78fd74a.
